# Longitudinal MRI study after carbon ion and photon irradiation: shorter latency time for myelopathy is not associated with differential morphological changes

**DOI:** 10.1186/s13014-021-01792-8

**Published:** 2021-03-31

**Authors:** Thomas Welzel, Alina L. Bendinger, Christin Glowa, Inna Babushkina, Manfred Jugold, Peter Peschke, Jürgen Debus, Christian P. Karger, Maria Saager

**Affiliations:** 1 Heidelberg Institute for Radiation Oncology (HIRO) and National Center for Radiation Research in Oncology (NCRO), Heidelberg, Germany; 2 Department of Radiation Oncology and Radiotherapy, University Hospital of Heidelberg, Heidelberg, Germany; 3 Department of Medical Physics in Radiation Oncology (E040), German Cancer Research Center (DKFZ), Im Neuenheimer Feld 280, 69120 Heidelberg, Germany; 4 Core Facility Small Animal Imaging Center, German Cancer Research Center (DKFZ), Heidelberg, Germany; 5 Clinical Cooperation Unit Radiation Therapy, German Cancer Research Center (DKFZ), Heidelberg, Germany

**Keywords:** Cervical spinal cord, Late radiation effects, Carbon ion irradiation, Myelopathy, Magnetic resonance imaging

## Abstract

**Background:**

Radiation-induced myelopathy is a severe and irreversible complication that occurs after a long symptom-free latency time if the spinal cord was exposed to a significant irradiation dose during tumor treatment. As carbon ions are increasingly investigated for tumor treatment in clinical trials, their effect on normal tissue needs further investigation to assure safety of patient treatments. Magnetic resonance imaging (MRI)-visible morphological alterations could serve as predictive markers for medicinal interventions to avoid severe side effects. Thus, MRI-visible morphological alterations in the rat spinal cord after high dose photon and carbon ion irradiation and their latency times were investigated.

**Methods:**

Rats whose spinal cords were irradiated with iso-effective high photon (n = 8) or carbon ion (n = 8) doses as well as sham-treated control animals (n = 6) underwent frequent MRI measurements until they developed radiation-induced myelopathy (paresis II). MR images were analyzed for morphological alterations and animals were regularly tested for neurological deficits. In addition, histological analysis was performed of animals suffering from paresis II compared to controls.

**Results:**

For both beam modalities, first morphological alterations occurred outside the spinal cord (bone marrow conversion, contrast agent accumulation in the musculature ventral and dorsal to the spinal cord) followed by morphological alterations inside the spinal cord (edema, syrinx, contrast agent accumulation) and eventually neurological alterations (paresis I and II). Latency times were significantly shorter after carbon ions as compared to photon irradiation.

**Conclusions:**

Irradiation of the rat spinal cord with photon or carbon ion doses that lead to 100% myelopathy induced a comparable fixed sequence of MRI-visible morphological alterations and neurological distortions. However, at least in the animal model used in this study, the observed MRI-visible morphological alterations in the spinal cord are not suited as predictive markers to identify animals that will develop myelopathy as the time between MRI-visible alterations and the occurrence of myelopathy is too short to intervene with protective or mitigative drugs.

## Introduction

Carbon ions are being increasingly investigated for radiotherapy in clinical trials [1], as they exhibit physical advantages, such as a finite range in tissue leading to highly conformal dose distributions, as well as biological advantages, associated with their increased linear energy transfer (LET) resulting in a higher relative biological effectiveness (RBE) in the target volume [2]. This makes them a promising candidate for irradiation of radioresistant and critically located tumors. However, despite the increased accuracy of carbon ions, the exposure of tumor surrounding normal tissue to high irradiation doses cannot be avoided. To assure patients’ safety, it is therefore of utmost importance to investigate the biological effect of carbon ions on normal tissue complication in comparison to conventionally used photons.

Late delayed injury like irradiation-induced myelopathy is a severe and irreversible complication that may occur 6 months to 2 years after irradiation [3]. Affected patients suffer from numbness, dysesthesias, and incontinence. The neurological deficit develops progressively eventually leading up to quadriplegia in at least 50% of patients [4]. On a histological level, irradiation-induced myelopathy is characterized by demyelination, vascular abnormalities, and necrosis in the white matter [5]. However, the primary target cell whose death leads to the irradiation-induced necrosis is not yet identified and the hypotheses that the vasculature and endothelial cells are the main cause for the development of late effects is still being investigated [5, 6].

The rat cervical spinal cord is a well-established model for the investigation of irradiation effects on normal tissue [7–11]. The biological endpoint irradiation-induced myelopathy can be well detected as paresis of the animals’ forelimbs. The morphological changes accompanying irradiation-induced myelopathy at different pre-designated time points after irradiation have been investigated on a histological level for single exposures of the thoracic and lumbar cord [12–14]. However, the results of these studies might not be representative for fractionated treatments used in clinical routine.

Patients suffering from delayed myelopathy show widening of the spinal cord and increased signal intensities in T2-weighted magnetic resonance (MR) images. Additionally, patients often show abnormal intramedullary contrast enhancement [4]. Therefore, we chose MR imaging, which allows repeated and non-invasive measurements, to systematically investigate the morphological tissue alterations that precede neurological disorders. In this way, disease progression may be monitored individually per animal and findings may be transferred to patients. So far, however, only few MRI studies have been performed on the pathogenesis of irradiation-induced myelopathy in the rat spinal cord [15, 16].

Regarding fractionated carbon ion irradiations of the cervical spinal cord, alterations preceding neurological disorders have not yet been investigated. A better understanding of the processes preceding clinical symptoms could help to find an adequate time window for medical interventions to prevent or mitigate radiation-induced myelopathy [17–20].

In this study, the longitudinal development of morphological and neurological alterations preceding radiation-induced myelopathy was observed and analyzed qualitatively by MRI after fractionated iso-effective irradiations with photons or carbon ions, respectively.

## Materials and methods

### Animals

A total of 22 young adult female (242 ± 34 g) Sprague–Dawley rats (Charles River, Sulzfeld, Germany) were used. Animals were randomly distributed into three groups: (1) 8 rats were irradiated with carbon ions, (2) 8 rats were irradiated with photons, and (3) 6 rats served as non-irradiated controls. Animals were kept under the standard laboratory conditions at the German Cancer Research Center (DKFZ, Heidelberg, Germany). All experiments were approved by the governmental review committee on animal care.

### Irradiation setup

The rat cervical spinal cord was irradiated with a single field of either carbon ions or photons. A detailed description of the dosimetry and the experimental set up can be found in [7, 8]. For irradiation with both modalities, rats were anesthetized with a mixture of 4 vol% Sevoflurane (Abbott, Wiesbaden, Germany) and oxygen at 2 l/min and animals were irradiated in a hanging position in a customized holding device [7]. The rat spinal cord was irradiated from ventral to dorsal with a horizontal beam (field size of 10 × 15 mm^2^) that covered the cervical segments C1–C6 with C3 being in the center and C1 and C6 being located at the field edge.

Carbon ion irradiations were performed at the Heidelberg Ion-Beam Therapy Center (HIT, Heidelberg, Germany). The cervical spinal cord was positioned in the middle of a 1 cm spread-out Bragg peak (SOBP) with a dose-averaged LET of 91 keV/µm (80–104 keV/µm) and was irradiated with a total dose of 23 Gy given in 6 fractions (3.83 Gy/fraction) on consecutive days. This dose was selected to reach 100% complication probability, i.e. myelopathy within 300 days in all animals [8]. Photon irradiations (6 meV, Artiste, Siemens, Erlangen) were performed with the respective iso-effective total dose of 61 Gy given in 6 fractions (10.167 Gy/fraction) on consecutive days to also reach 100% complication probability [8]. Six fractions were chosen for irradiation to get closer to a typical patient treatment schedule, and the iso-effective doses for photons and carbon ions and the respective RBE for the endpoint radiation-induced myelopathy within 300 days were known from a previous study [8].

### MRI follow-up

**Fig. 1:**
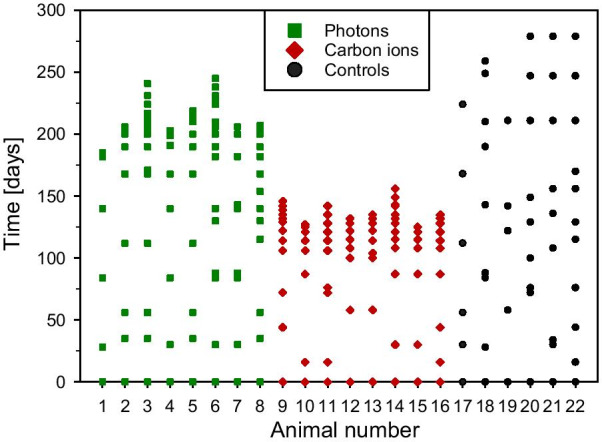
Time points of MRI of non-irradiated controls (black), and animals irradiated with photons (green) or carbon ions (red), respectively. Animals were first checked monthly for morphological alterations. As soon as an alteration occurred, the respective rat was monitored in shorter time intervals

Animals underwent MRI before irradiation and were then imaged on a monthly basis to detect morphological alterations. As soon as an alteration occurred, the respective rat was monitored in shorter time intervals (Fig. 1).

MRI measurements were performed on a 1.5 T whole-body MR-scanner (Symphony, Siemens, Erlangen, Germany) using an in-house built small animal coil. Rats were anesthetized by inhalation anesthesia (Isoflurane, Baxter, 2.5 vol% in 1.2 l/min oxygen). During each MRI session, sagittal and axial T2-weighted images (4000/109 ms [repetition time/echo time], voxel size 0.3 × 0.3 × 1.0 mm) were acquired per animal to assess morphological changes such as edema formation. Additionally, T1-weighted images (600/14 ms [repetition time/echo time], voxel size 0.2 × 0.2 × 1.0 mm) were recorded before and after contrast agent injection to examine the onset of the blood spinal cord barrier (BSCB) disruption. The contrast agent (0.2 mmol/kg Magnevist®, Bayer, in 0.9% NaCl) was injected intravenously as a bolus via the tail vein and animals were imaged 5 min after injection.

### Follow up and endpoints

After irradiation, rats were checked weekly for neurological alterations, weight, and general condition. Neurological alterations were checked by letting the rats walk on a flat surface and scoring their movements according to Debus et al. [7]. The final biological endpoint was radiation-induced myelopathy (paresis II) within 300 days meaning that both forelimbs showed signs of paralysis [7], such as regular dragging of the foot with palmar flexion or dragging of extended forelegs. As soon as this was observed in an animal, it was scored as having paresis II, a final MRI measurement was performed, and the animal was sacrificed. Before the animals reached the final endpoint, they showed various morphological alterations (see “Image evaluation”) and paresis I (at least one forelimb showed signs of neurological dysfunction [7]). The latency times, defined as the time between the day of the first fraction of irradiation and the occurrence of the respective morphological and neurological alterations after irradiation, were recorded for each animal.

### Image evaluation

Two blinded readers (T.W., M.S.) performed qualitative visual inspections of the MR-images with a consensus reading of pathologies to determine the latency times for the morphological alterations. For this, the axial and sagittal T2-weighted images as well as the sagittal T1-weighted images (native and post contrast agent) were used.

Native T1-weighted images were used to identify bone marrow conversion. To examine the onset of loss of BSCB integrity, native and enhanced T1-weighted images were compared. The T2-weighted images were used to identify edema and the dilatation of the *canalis centralis* (syrinx). All latency times, i.e. the time between the first fraction and the occurrence of MR visible morphological alterations, were recorded.

### Histology

When animals reached paresis II they were sacrificed by an overdose of *i.p.* injected Xylazine/Ketamine (100 µl/kg each). Animals were perfused with a mixture of 4% paraformaldehyde (PFA) in phosphate buffered saline before the cervical spinal cord C1–C6 were dissected and fixated in PFA. After dehydration in ethanol the segments C1–C6 were embedded in paraffin with an axial orientation.

All histological stainings were performed on 9 µm thick paraffin sections that were deparaffinized and rehydrated before staining. For all immunohistochemistry stainings antigen retrieval in sodium citrate buffer (pH 6) was performed for 30 min at 95 °C to unmask antigen sites. At the end, sections were dehydrated and mounted in Eukitt.

For qualitative examination of the extent of demyelination, paraffin sections were stained with Luxol fast blue which binds to the lipoproteins of myelin [21] in combination with hemalaun/eosin (H&E). Sections were incubated in 0.1% Luxol blue at 50 °C over night, and washed in 95% ethanol, and differentiated in 0.05% lithium carbonat and 70% ethanol the next day. Afterwards, sections were counterstained with hematoxylin and eosin.

To study the degree of blood vessel permeability, extravagated endogenous serum albumin was immunohistochemically visualized. Endogenous peroxidase activity was blocked with 3% H_2_O_2_. After antigen retrieval, sections were incubated overnight at 4 °C with the primary antibody against albumin (Acris, 1:6000 diluted in 3% bovine serum albumin in TBS-T) followed by incubation with the secondary antibody (Abcam, 1:500, horse raddish peroxidase) for 30 min at room temperature. 3,3′-diaminobenzidine was used as chromogen (Vector Laboratories, Burlingame, CA, US). In the end, sections were counterstained with 1% Nissl in sodium acetate buffer.

To assess the degradation of the blood spinal cord barrier, sections were stained for the endothelial barrier antigen (EBA), a protein that is exclusively expressed on the luminal surface of non-fenestrated central nervous system blood vessels [22], in combination with the endothelial marker CD34. Autofluorescence was quenched by 30 min incubation with 0.1% Sudan Black B (Sigma-Aldrich®, Missouri, US). After blocking in 10% normal goat serum, sections were incubated with the primary antibodies monoclonal mouse anti-EBA SMI 71 (1:500, Biolegend, San Diego, Ca, US) and recombinant rabbit anti-CD34 antibody (1:1000, abcam, Cambridge, UK) in 3% bovine serum albumin in TBS-T at 4 °C over night. For detection, sections were incubated with the fluorescent-coupled secondary antibodies goat anti-rabbit Alexa Flour 555 (1:1000, Molecular Probes®, Invitrogen AG, Carlsbad, USA) and goat anti-mouse Alexa Fluor 488 (1:1000, Molecular Probes®) at room temperature for 30 min each. Sections were counter stained with DAPI and mounted with Fluoromount-G.

### Statistical analyses

The difference in the latency times per morphological and neurological alterations between the irradiation modalities was tested by a two-sided student t-test with a significance level of 0.05. The longitudinal difference of the latency times within one irradiation modality was tested by one way repeated measure ANOVA. All statistical testing was performed in SigmaPlot 14.0 (Systat Software Inc., San Jose, CA, USA).

## Results

### Skin alterations

Irradiation procedures as well as MRI measurements were well tolerated by all animals. None of the animals died due to acute radiation toxicity or contrast agent intolerance. Acute radiation toxicity developed within the first 4 weeks after photon treatment and included a slight or complete hair loss as well as moist desquamation restricted to a small focal region at the ventral and dorsal side of the rats’ throat. Skin alterations were less severe at the dorsal than at the ventral side. Due to the narrow SOBP, no skin alterations were observed after carbon ion irradiation.

### Latency times

**Fig. 2:**
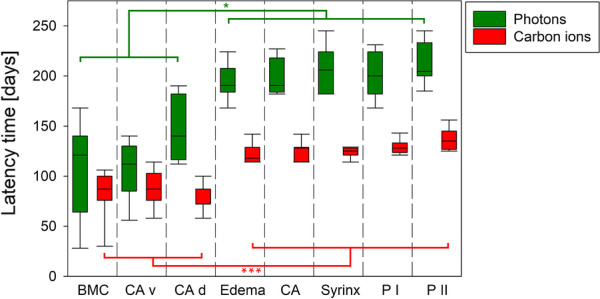
Latency times until occurrence of morphological alterations and neurological distortions after irradiation with photons (green) or carbon ions (red). For both irradiation modalities, the morphological alterations outside the spinal cord (BMC, CA v, CA d) occurred significantly earlier than morphological alterations inside the spinal cord (edema, CA, syrinx) and the start of neurological distortions (P I, P II). Box plots (25%/75%) show median (solid line) with 10%/90% percentiles (whiskers). BMC = bone marrow conversion, CA v/d = contrast agent accumulation in the ventral and dorsal musculature outside of the spinal cord, respectively, CA = contrast agent accumulation inside the spinal cord, P I (paresis I) = at least one forelimb showed signs of neurological dysfunction, P II (paresis II) = both forelimbs showed signs of paralysis. *p < 0.05, ***p < 0.001. *Note*: Statistical comparisons between MRI endpoints outside the spinal cord (BMC, CA v/d) or MRI endpoints inside the spinal (edema, CA, syrinx) cord plus neurological endpoints (P I, P II) were non-significant while any combination between these two groups of endpoints were significant

The latency times detected for the different MR-visible morphological alterations and neurological distortions after irradiation with photons or carbon ions are summarized in Table 1 and visualized in Fig. 2. All animals reached the biological endpoint paresis II within 300 days.

**Table 1:** Mean latency times (± standard deviation) of morphological alterations and neurological distortions after photon and carbon ion irradiation

	Time of onset of morphological and/or neurological alterations [days after irradiation]							
	BMC	CA v	CA d	Edema	CA	Syrinx	P I	P II
Photons	108 ± 47	107 ± 29	**149 ± 32**	**194 ± 17**	**198 ± 18**^**b**^	**206 ± 23**^**a**^	**199 ± 22**^**a**^	**212 ± 21**
^12^C-ions	84 ± 24	88 ± 18	**81 ± 13**	**122 ± 10**	**125 ± 10**^**c**^	**124 ± 5**	**129 ± 7**	**137 ± 11**

Bold printed latency times are significantly different (*p* < 0.001) between photon and carbon ion irradiated animals

*BMC* bone marrow conversion, *CA v/d* contrast agent enhancement in the ventral/dorsal musculature outside the spinal cord, respectively, *CA* contrast agent enhancement inside the spinal cord, *P I (paresis I)* at least one forelimb showed signs of neurological dysfunction, *P II (paresis II)* both forelimbs showed signs of paralysis

^a^7 out of 8 rats

^b^3 out of 7 animals showed CA without paresis I

^c^5 out of 8 animals showed CA without paresis II

**Fig. 3:**
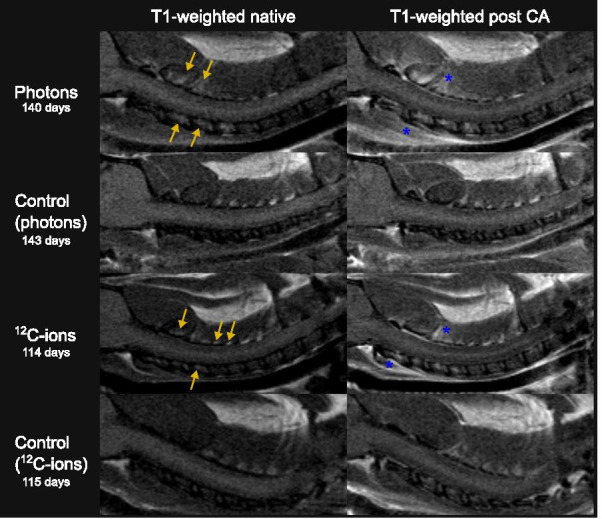
Exemplary MR-images of irradiation-induced morphological alterations outside the spinal cord compared to respective non-irradiated age-matched controls. Native T1-weighted images of the irradiated rat cervical spinal cord (C1–6) show a hyperintense signal in the vertebral body (yellow arrows) due to bone marrow conversion. T1-weighted images post contrast agent (CA) injection of irradiated animals show CA accumulation in the musculature dorsal and ventral to the spinal cord (blue asterisks) compared to the native T1-weighted. Age-matched control animals of each treatment arm accumulated no CA

The first irradiation-induced morphological alterations that occurred were located outside the spinal cord (Fig. 3). Bone marrow conversion (BMC) within the cervical vertebral bodies C1–C4 appeared as hyperintense signal in the native T1-weighted MR images. Next, contrast agent started to leak through the vasculature and accumulated in the ventral and dorsal musculature surrounding the cervical spinal cord. While the onset of contrast agent accumulation in the ventral muscle was rather concurrent to the first signs of BMC per modality, the contrast agent accumulation was significantly delayed in the dorsal muscle in photon irradiated animals as compared to carbon ion irradiated animals. The contrast agent accumulated to a greater extend after photon than after carbon ion irradiations.

**Fig. 4:**
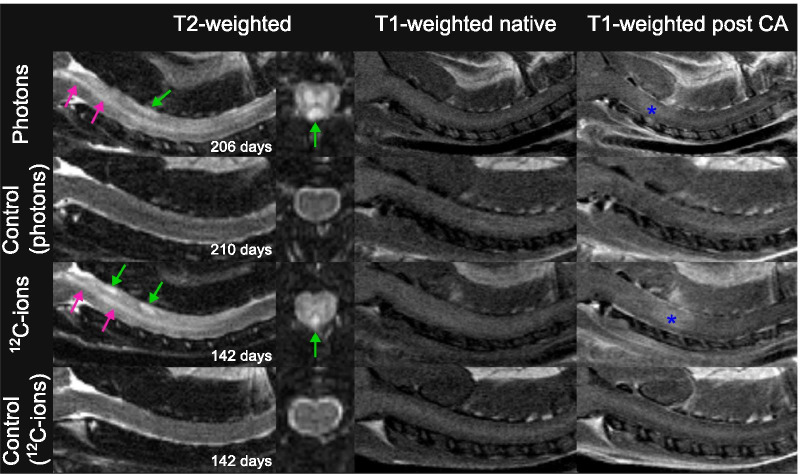
Exemplary MR-images of irradiation-induced morphological alterations inside the spinal cord of two animals with paresis II after irradiation with photons or ^12^C-ions and their age-matched controls. Syrinx (pink arrows) and edema (green arrows) are indicated by hyperintense signals in the T2-weighted images of irradiated animals. T1-weighted images after contrast agent (CA) injection show CA accumulation in the spinal cord (blue asterisks) compared to native T1-weighted images. No CA accumulation was detected in the spinal cord of the age-matched non-irradiated controls

In the longitudinal course, morphological alterations such as the presence of edema in the spinal cord, a dilatation of the *canalis centralis* (syrinx), and accumulation of contrast agent alongside the spinal cord were observed concurrently, but significantly later than for the spinal cord external alterations in both irradiation modalities (Table 1, Figs. 2, 4). Out of the seven photon-irradiated animals that showed paresis I, three showed contrast agent accumulation in the spinal cord before developing signs of paresis. For carbon ion irradiations, the same morphological alterations were observed (Fig. 4), but the latency times were significantly shorter compared to photon irradiations. In five out of eight animals, contrast agent accumulation in the spinal cord was detected before paresis I had developed.

Next, animals developed neurological deficits that rapidly impaired to paresis I and finally to the endpoint paresis II. One animal of the photon treated group went so rapidly to paresis II that paresis I could not be detected separately. Three out of seven animals developed paresis I prior to a syrinx. All carbon ion irradiated animals showed edema, syrinx and contrast agent accumulation along the spinal cord when they reached the endpoint paresis II. T2-weighted images revealed that at this point edema and syrinx had spread along the irradiated part of C1–C6.

The biological endpoint paresis II occurred at 212 ± 21 days after photon and 137 ± 11 days after carbon ion irradiation. At these time points, the above described morphological changes had intensified. Edema and syrinx had spread further in cranial and caudal directions outside the irradiation field.

Overall, the sequence of morphological and neurological alterations was the same after photon and carbon ion irradiation. However, after the time point of dorsal accumulation of contrast agent, the latency times for the endpoints were significantly shorter for carbon ions than for photons. There was a large spread in latency times between the individual animals per irradiation group.

After photon irradiation, the mean time interval between the first morphological alteration (edema) and paresis I was 4 ± 6 days, after carbon ion irradiation it was 7 ± 5 days.

### Histology

Histological examination of animals with paresis II exhibited comparable tissue damages after photon and carbon ion irradiation.

**Fig. 5:**
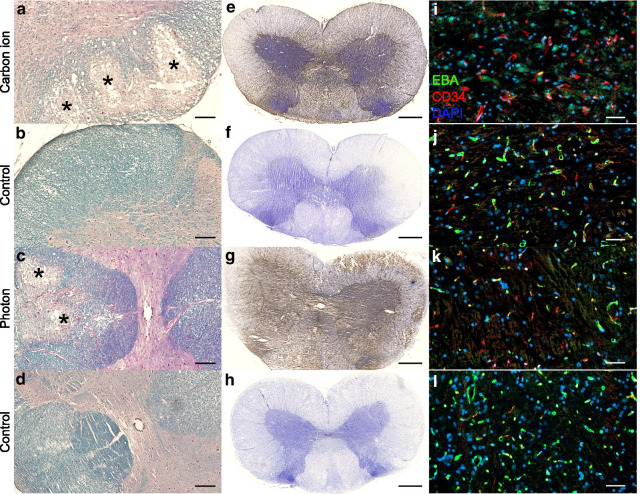
Representative axial histological images of carbon ion and photon irradiated animals with paresis II and their respective age-matched controls. **a**–**d** Luxol fast blue staining revealing focal demyelination and necrosis (asterisks) in the *S. alba* of irradiated animals while control animals showed dense tissue structure (scale bar 200 µm). **e**–**h** Endogenous albumin extravasation, represented by brown precipitation, indicates the breakdown of the BSCB. **i**–**l** Irradiated segments show a severe decrease in intact blood vessels (CD34, red) and breakdown of the BSCB (EBA, green). Overlap of CD34 and EBA appears yellow. Cell nuclei were counter stained with DAPI (scale bar 50 µm)

Luxol fast blue staining revealed focal demyelination and severe necrosis in the *S. alba*. Focal necroses were located in the right and left *F. lateralis* and the *F. posterior* (Fig. 5a–d). Necrosis was more severe after carbon ion irradiation than after photons. Local bleedings occurred in the *S. grisea* of affected segments. The overall structure remained visually intact.

The extravasation of endogenous albumin, representing the breakdown of the BSCB, was extensive and comparable between photon and carbon ion irradiated spinal cords (Fig. 5e, g). All segments C1–C6 were affected even though the outer segments showed mostly albumin extravasation in the posterior part of the white matter and around the *canalis centralis*. No albumin had extravasated in the age-matched control animals (Fig. 5f, h).

Segments with focal demyelination exhibited hardly any intact vessels as EBA expression was extensively decreased in both, the *S. alba* and *S. grisea*, in those segments of irradiated animals (Fig. 5i–l). CD34 revealed vessel dilatation in the grey matter of irradiated animals.

## Discussion

During radiotherapy, tumor surrounding normal tissue is often partially or fully exposed to high doses, which can lead to irreversible side effects. For the spinal cord, radiation-induced myelopathy represents a delayed late effect, which is characterized by a long symptom free latency period of up to 2 years followed by a sudden occurrence of neurological deficits in the patient [3]. As carbon ions are increasingly investigated in clinical trials with focus on brain and head and neck tumors, their effect on normal tissue of the central nervous system needs to be explored. For this, we performed a longitudinal MRI study to characterize the temporal development of irradiation-induced myelopathy in the rat during the symptom free latency period after fractionated iso-effective irradiation with photons or carbon ions and to identify MR-visible morphological alterations that could serve as predictive signs of late radiation injury.

### Acute reactions

While the photon and carbon ion doses to the spinal cord were selected to be iso-effective, acute skin reactions such as slight or complete hair loss and moist desquamation of the ventral and dorsal side of the rats’ throat, occurred only after photon irradiation. Therefore, the differential skin reactions after both irradiation modalities cannot be attributed to RBE effects, but rather to differential depth dose profiles. Unlike photons, carbon ions deposit their dose within a narrow SOBP, which physically spares the skin. This side observation demonstrates the advantage of the high dose conformity that can be reached with carbon ions. This is similar in patients and may be important for their quality of life [23]. Beyond acute reactions, the exposure of smaller volumes with carbon ions may be of importance for tissues exhibiting a distinct volume effect (e.g. brain). As normal tissue tolerance is entangled with the volume effect, conformal irradiations are generally of advantage. Acute skin reactions as observed in this study may also be used as a model for investigating acute normal tissue reactions after irradiation [24–26].

### Fixed sequence of MR-visible morphological alterations and neurological distortions

Irradiation with photons and carbon ions at iso-effective doses for the endpoint myelopathy within 300 days, induced a fixed sequence of MR-visible morphological alterations that precede paresis II. First morphological alterations occurred in the structures surrounding the spinal cord (BMC, CA v/d). Bone marrow conversion is observed due to fatty replacement of the marrow after irradiation [27] and it is non-reversible above a threshold of approximately 30–40 Gy [28]. The longer latency time until contrast agent accumulated in the musculature dorsal to the spinal cord after photons but not after carbon ions underlines the independence of carbon ion effectiveness of tissue type [29]. Significantly later than the external alterations, morphological changes appeared inside the spinal cord, including edema, syrinx and the breakdown of the BSCB. The latter was indicated by contrast agent accumulation in the spinal cord and by the detection of extravasated endogenous albumin in histological stainings. The observed alterations are in accordance with findings in patients that had developed myelopathy [30, 31].

In our study, iso-effective treatment with the two irradiation modalities only led to differences in latency times: the sequence of morphological and neurological alterations started significantly earlier after carbon ions than after photons. A shorter latency time for irradiation induced myelopathy was also found in other high-LET studies [7, 8, 13, 32, 33]. High LET radiation such as carbon ions, induces clustered DNA damage and reduces the cell’s repair capacity significantly [34], which may be the main cause for the shorter latency time. Furthermore, the latency time for irradiation induced myelopathy has also been shown to decrease with increasing LET, fractionation number, and dose [35].

Interestingly, the time interval between first MRI-visible morphological alterations (edema) and paresis I and II was comparable for both irradiation modalities. This is also in accordance with the histological results, which showed comparable tissue and vascular degradation after irradiation with both modalities. These findings indicate that the progresses that take place on a morphological and functional level visible in MRI as well as in histology are comparable after low and high LET irradiation. Similar histological results were reported by Okada et al. [13] after single fraction irradiation of murine thoracic and lumbar spinal cord with carbon ions and photons.

### Predictive markers for medicinal interventions

From clinical experience, radiation-induced myelopathy develops suddenly after a long symptom-free period and once neurological deficits appear, these are usually irreversible. Therefore, it is of great interest to non-invasively determine preceding morphological changes that could serve as predictive markers to potentially initiate medicinal interventions that might prevent or mitigate clinical symptoms.

In this regard, the vascular hypothesis assumes that radiation-induced vascular damage and subsequent ischemia leads to white matter necrosis [5]. The contrast agent accumulation in the spinal cord, as measured by MRI, marks the breakdown of the BSCB of the vasculature, and hence, would be a promising candidate for such a predictive marker. In support of this, we found contrast agent accumulation in the spinal cord before paresis I in half of the animals, suggesting that the breakdown of the BSCB is a prerequisite for the development of neurological symptoms. However, the development of myelopathy is rapid and the mean time between the first detection of an edema and contrast agent accumulation and the onset of paresis I and II was less than 1 week. Unfortunately, this time window is too narrow for successful medicinal intervention. In contrast, the CA accumulation in the ventral musculature of the spinal cord was observed significantly earlier than paresis I (94 ± 37 days earlier for photon irradiation and 42 ± 18 days earlier for carbon ion irradiation). Whether this can be regarded as an early indication for the development of myelopathy or whether it resembles an independent reaction of the musculature’s vasculature requires further experiments with lower irradiation doses. Furthermore, experiments with lower irradiation doses are required to find out whether the observed morphological alterations are exclusive precursors of severe neurological dysfunctions.

Nevertheless, with regard to the low sensitivity of the used MRI scanner, investigation of the molecular pathways, e.g. from blood samples, prior to the MR visible alterations appear necessary to identify starting points for medicinal interventions. As an example, administration of ACE-inhibitor after irradiation has been reported to mitigate radiation-induced myelopathy in healthy central nervous system tissue [17].

## Conclusion

The development of myelopathy is free of symptoms over a long time, but then occurs rather rapidly and irreversible. This observational study gives first insights into the time course of morphological alterations in MRI preceding radiation-induced myelopathy. We found a modality-independent fixed sequence of morphological and neurological alterations that has a significantly shorter latency time after carbon ion than after photon irradiation despite the iso-effective doses that were used. However, at least in the animal model used in this study, the observed MR-visible morphological alterations in the spinal cord are not suited as predictive markers to identify animals that will develop myelopathy as the time between MR-visible alterations and the occurrence of myelopathy is too short to intervene with protective or mitigative drugs.

## Data Availability

The datasets analyzed during the current study are available from the corresponding author on reasonable request.

## Abbreviations

^12^C-ions: Carbon ions
BSCB: Blood spinal cord barrier
CA: Contrast agent
CA d: Contrast agent accumulation in the dorsal musculature of the spinal cord
CA v: Contrast agent accumulation in the ventral musculature of the spinal cord
EBA: Endothelial barrier antigen
H&E: Hemalaun/eosin
LET: Linear energy transfer
MRI: Magnetic resonance imaging
P I: Paresis I
P II: Paresis II
RBE: Relative biological effectiveness
SOBP: Spread-out Bragg peak
